# Placental Transfer of Perfluoroalkyl Substances and Associations with Thyroid Hormones: Beijing Prenatal Exposure Study

**DOI:** 10.1038/srep21699

**Published:** 2016-02-22

**Authors:** Lin Yang, Jingguang Li, Jianqiang Lai, Hemi Luan, Zongwei Cai, Yibaina Wang, Yunfeng Zhao, Yongning Wu

**Affiliations:** 1 Key Laboratory of Food Safety Risk Assessment, Ministry of Health and China National Center for Food Safety Risk Assessment, No. 7, Panjiayuannanli, 100021, Beijing, China; 2 National Institute of Nutrition and Health, Chinese Centre for Disease Control and Prevention, China; 3 State Key Laboratory of Environmental and Biological Analysis, Department of Chemistry, Hong Kong Baptist University, Kowloon Tong, Hong Kong SAR, China

## Abstract

Perfluoroalkyl substances (PFASs) have been detected in wildlife and human samples worldwide. Toxicology research showed that PFASs could interfere with thyroid hormone homeostasis. In this study, eight PFASs, fifteen PFAS precursors and five thyroid hormones were analyzed in 157 paired maternal and cord serum samples collected in Beijing around delivery. Seven PFASs and two precursors were detected in both maternal and cord sera with significant maternal-fetal correlations (r = 0.336 to 0.806, all *P* < 0.001). The median ratios of major PFASs concentrations in fetal versus maternal serum were from 0.25:1 (perfluorodecanoic acid, PFDA) to 0.65:1 (perfluorooctanoic acid, PFOA). Spearman partial correlation test showed that maternal thyroid stimulating hormone (TSH) was negatively correlated with most maternal PFASs (r = −0.261 to −0.170, all *P* < 0.05). Maternal triiodothyronin (T3) and free T3 (FT3) showed negative correlations with most fetal PFASs (r = −0.229 to −0.165 for T3; r = −0.293 to −0.169 for FT3, all *P* < 0.05). Our results suggest prenatal exposure of fetus to PFASs and potential associations between PFASs and thyroid hormone homeostasis in humans.

Perfluoroalkyl substances (PFASs) have been extensively used in a wide range of industrial and consumer applications such as surfactants, lubricants, photographic emulsifiers, paints, fire-fighting foams and food packaging due to their unique hydrophobic and lipophobic nature^1^. Food and water contamination, dermal contact, household dust and air inhalation are different pathways of human exposure to those compounds^2^,^3^. Multiple studies showed ubiquitous detection of PFASs in wildlife and human samples^4^,^5^,^6^,^7^,^8^,^9^,^10^,^11^. Although perfluorooctane sulfonate (PFOS) and perfluorooctane sulfonyl fluoride were listed as “restricted use” compounds in Annex B of the Stockholm Convention on persistent organic pollutants in 2009^12^, relatively large amounts of these chemicals are still manufactured and used in China^13^,^14^,^15^.

Experimental studies have found that PFASs can interfere with thyroid hormone homeostasis. In rats, single-dose exposure to PFOS transiently increased free thyroxine (FT4) and decreased thyroid-stimulating hormone (TSH) in 6 h, followed by decreased thyroxine (T4) and triiodothyronine (T3)^16^. Perfluorooctanoic acid (PFOA) and PFOS short-term treatment in rats both resulted in lowered T4 and T3 levels^17^. And longer-term exposure to PFOS reduced T4 level^18^ or both T4 and T3 levels^19^. PFOS treatment in pregnant rats caused reduction of T4 and T3 levels without a compensatory rise of TSH^20^,^21^, and decreased serum levels of T4 in the offspring^21^,^22^,^23^. Studies in monkeys showed lowered T3 and free T3 (FT3) levels after exposure to PFOS^24^ and ammonium salt of PFOA^25^. In human studies, associations between PFOA or PFOS and thyroid disease have been found in general population^26^ and in highly exposed Mid-Ohio Valley community children, adults and workers^27^,^28^,^29^. However, the results of many other population-based researches on individual thyroid hormones disrupting potencies of PFASs were inconsistent^30^,^31^,^32^,^33^,^34^,^35^,^36^. Null associations between PFASs and thyroid hormones have been also reported^37^,^38^.

Several studies have examined the effects of PFASs on thyroid status among pregnant women. For example, the concentrations of PFASs in a case-control study of Canadian pregnant women were found not associated with hypothyroxinemia^39^. However, Kim *et al.* in South Korea have found the significant negative correlations between maternal PFASs and fetal T4 and T3^40^. Two studies in Denmark and Taiwan reported by Wang *et al.* also showed the interference of maternal PFASs on thyroid hormone homeostasis in pregnant women and fetuses^41^,^42^. Latest researches in Canada and Norway have found positive associations between maternal PFASs and maternal TSH during the second trimester^43^,^44^.

Thyroid hormones throughout gestation are essential for the growth and neurodevelopment of fetuses^45^,^46^,^47^,^48^. In one latest study, the prenatal exposure of fetuses to PFASs was even found associated with decreased IQ test scores in children^49^. The potential effect of PFAS prenatal exposure on thyroid hormone homeostasis should be concerned for the health of pregnant women and their fetuses. In this study, eight PFASs, fifteen PFAS precursors and five thyroid hormones (TSH, T3, T4, FT3 and FT4) were measured in 157 paired maternal and cord serum samples collected in Beijing around delivery. Our aims include: 1) determine placental transfer of PFASs; 2) investigate the potential associations between PFAS concentrations and thyroid hormone levels in maternal and cord serum.

## Results

### Participant characteristics

The characteristics of the 157 pregnant women and their neonates are shown in Table 1. Participants averaged 30 years old, had a mean prepregnancy body mass index (BMI) of 21.1 kg/m^2^. Most women were primiparous and more than half of them had a cesarean section for delivery. All participants have no experience of occupational exposure to PFASs and generally did not smoke or consume alcohol during pregnancy. The number of the male infants was more than the female ones. The mean birth weight of neonates was 3447 g and the mean birth length was 50.5 cm.

### Concentrations of PFASs and thyroid hormones in maternal and cord sera

Among the eight PFASs and fifteen PFAS precursors, compounds detected in less than 30% of samples were not considered further. Perfluorohexanesulfonate (PFHxS), PFOS, PFOA, perfluorononanoic acid (PFNA), perfluorodecanoic acid (PFDA) were detected in all samples, and perfluoroundecanoic acid (PFUnA) were detected in > 95% maternal and cord serum samples (Table 2). PFOS, followed by PFOA, was the dominant PFAS contaminant with highest level and Perfluorododecanoic acid (PFDoA) was the lowest one in both maternal and cord serum samples. 6:2 fluorotelomer sulfonates (6:2 FTS) and *N*-methyl perfluorooctanesulfonamidoacetate (NMeFOSAA) were the only two PFAS precursors detected in considerable serum samples. The concentration of 6:2 FTS was higher than NMeFOSAA, but both at the low level of pg/mL. All PFASs and precursors had significant maternal-fetal correlations (r = 0.336 to 0.806, all *P* < 0.001). The median ratios of major PFAS concentrations in fetal versus maternal serum were from 0.25:1 to 0.65:1 (Table 2). Besides, in maternal serum, PFNA, PFDA, and PFUnA were highly correlated (r ≥ 0.832, all *P* < 0.001) and they were all moderately correlated with PFOS, PFOA, and PFDoA (r = 0.374 to 0.715, all *P* < 0.001) (see Supplementary Information, Table S1). The composition profiles of major PFASs in maternal and cord serum were presented in the Supplementary Information, Fig. S1.

The concentrations of thyroid hormones in maternal and fetal serum were shown in Table 3. Among the five thyroid hormones, only T4 showed statistically significant but weak correlation between concentrations in maternal and cord serum (r = 0.260, all *P* = 0.001).

### Associations between PFASs and thyroid hormones in maternal and cord sera

In maternal serum, as shown in Table 4, FT3, FT4, T4 and T3 were negatively correlated with several PFASs but most of the relationships were no longer statistically significant after adjustment for major covariates. Only PFDoA remained the negative correlations with all five thyroid hormones (r = −0.301 to −0.160, all *P* < 0.05). However, TSH was negatively correlated with most maternal PFASs with- or without adjustment for covariates (r = −0.261 to −0.170, all *P* < 0.05). On the contrary, 6:2 FTS showed positive correlations with FT3, T3, FT4 and T4 after covariates adjustment (r = 0.160 to 0.205, all *P* < 0.05).

In cord serum (see Supplementary Information, Table S2), PFOS turned to be positively correlated with FT3, T3 and T4 after adjustment for covariates (r = 0.170 to 0.191, all *P* < 0.05). Similarly, the significance of positive correlations between PFDA with T3 and T4 and PFUnA with T4 appeared after adjustment.

The cross influence between maternal PFASs and fetal thyroid hormones was subsequently investigated (see Supplementary Information, Table S3). Maternal PFHxS was correlated with all fetal thyroid hormones but the statistical significance disappeared after adjustment for covariates. Only maternal PFOA still showed negative correlation with fetal FT3 (r = −0.169, *P* < 0.05) after adjustment.

On the other hand, Spearman test of fetal PFASs and maternal thyroid hormones provided many negative correlations outcomes (Table 5). Those negative correlations between maternal FT3, T3 with most fetal PFASs remained statistically significant after adjustment for covariates (r = −0.293 to −0.169 for FT3; r = −0.229 to −0.165 for T3, all *P* < 0.05). Maternal TSH was negatively correlated with fetal PFOS (r = −0.173, *P* < 0.05).

Besides adjusting for covariates selected from the multivariate analysis, we also tested the results with adjustment for covariates based on literature (Table S12)^40^,^41^,^42^. Results were presented in the Supplementary Information, Tables S8–S11. The significance and direction of the adjusted correlations were similar between the analyses using literature covariates and covariates selected by our multivariate analysis.

## Discussion

In these data, seven PFASs and two precursors were detected in both maternal and cord sera with significant maternal-fetal correlations and different placental transfer ratios. Maternal TSH was negatively correlated with most maternal PFASs and maternal T3, FT3 were negatively correlated with most fetal PFASs.

The concentrations of the major PFASs in our study were lower than most reported studies of populations in other countries^4^,^5^,^7^,^11^,^31^,^32^,^36^,^39^, but comparable with levels detected in other cities from China^6^,^50^ and in recent studies of populations from Norway^51^, South Korea^40^, 20–40 years old females from the United States^36^ and Denmark^52^. The highly correlated PFASs in maternal serum, especially PFNA, PFDA and PFUnA, indicated that these chemicals might have common sources of exposure in this area. The significant maternal-fetal correlations among detected PFASs and precursors suggested placental transfer of these compounds from mother to fetuses. In a study of fifty mother-infant pairs in Jiangsu province from China, the median ratios of PFASs concentrations in fetal versus maternal serum were 0.39:1, 0.54:1, 0.57:1, 0.73:1, 0.89:1 for PFDA, PFOS, PFNA, PFHxS and PFOA^50^. In comparison, placental transfer ratios in our study were smaller but having the similar sequence of compounds. Another study of seventy-one paired serum samples in Cincinnati from the United States reported the geometric means of serum concentration ratios between cord and maternal serum were least for PFOS (0.40:1), followed by PFHxS (0.59:1), PFNA (0.64:1) and PFOA (0.83:1)^53^, which had the consistent sequence of compounds compared with our results. The smaller value of ratios in our study could be due to the differences in sample size, characteristics of populations and maternal serum sampling time, namely, 1–2 day before delivery for our study and after delivery for the other two studies. The ratios of 6:2 FTS and NMeFOSAA were quite high and fluctuant with large standard deviations, which might result from their low rates of detection and fluctuant concentrations at a pg/mL level in both maternal and cord sera.

In maternal serum, TSH was found negatively correlated with most maternal PFASs. This result was different from previous studies in pregnant women^41^,^44^, which reported that women in higher PFOS exposure had increased TSH level (Table 6). However, the subjects of these studies were in second trimester, having a higher PFOS concentration in serum^41^,^44^ (Table 6). The similar negative correlation between PFASs and TSH was once reported in non-pregnant population, for example, PFOS was found negatively correlated with TSH in a study of Inuit adults, which was consistent with our result. Interestingly, they also had a much higher PFOS serum level (geometric mean: 18.28 ng/mL, n = 621) than our study^30^. In one animal study, Chang *et al.* found single dose of PFOS in adult rats could lead a transient increase in FT4 and decrease in TSH^16^. Nonetheless, the associations between PFASs and TSH in our study were not a transient observation. PFDoA was found to negatively correlate with all five thyroid hormones in maternal serum. The similar negative association between maternal PFDoA and maternal FT4 and T4 was also reported by a study of Taiwan pregnant women (n = 285) in third trimester^42^ (Table 6). Toxicology researches showed that PFOS could alter thyroid hormone biosynthesis and metabolism by inducing uridinediphosphate glucuronosyltransferase UGT1A1 and upregulating organic anion transporter OAPT2 and multidrug resistance–associated protein MRP2 to enhance hepatic uptake and metabolism of thyroid hormones in rats^18^,^54^. The longer chain PFDoA might have the same toxicology mechanism resulting in the negative relationships with thyroid hormones. Although PFDoA has the lowest concentration among the seven detected PFASs in maternal serum, former researches have reported that the toxicity increased with increasing carbon chain length in PFASs^55^,^56^,^57^. The longest PFDoA might have the same or even greater impact on thyroid hormones homeostasis despite of the low serum level. However, these negative correlations should be treated with caution for the relative lower detection rate of PFDoA (68%) compared to other PFASs in our study. Future researches with higher detection rate and larger sample size are in need to confirm these negative associations, as well as more in-depth experimental studies to clarify the mechanism of PFDoA thyroid hormones disruption. For the PFAS precursors, there is no literature or possible explanation for the positive correlations between 6:2 FTS with FT3, T3, FT4 and T4 found in our study.

The cord TSH level was much higher than maternal TSH level as former work reported^42^, which is attributed to labor and delivery. Thyroid hormone levels from cord blood may be affected by delivery stress such as labor pain, the duration of labor, uterotonic agents and higher TSH level was reported in cesarean section compared to vaginal delivery^58^. The main reason we choose the cord serum samples is that it is very suitable to explore the placental transfer of PFASs and thyroids, as the cord serum was collected just 1–2 days after the collection of maternal serum. Except the weak maternal-cord association of T4, the overall lack of correlations between thyroid hormones in maternal and fetal serum around delivery may be due to the increasing autonomy of the fetal thyroid axis as the pregnancy proceeds^59^. For example, only about 30% of fetal T4 originates from the maternal blood at birth^60^. With both sources from mothers and fetuses, the reason for the alteration in fetal thyroid hormones is complicated. However, the positive correlations between fetal PFOS with fetal T3 and T4 in our study were opposite to the negative correlation between fetal PFOS and fetal T4 found in Korean pregnant women (n = 44)^40^. But those negative correlations in that study were no longer significant after adjustment for covariates.

The influence of maternal PFASs on fetal thyroid hormones might include altering the placental transfer of thyroid hormones by competitive binding to transporter proteins. Weiss *et al.* reported that PFASs could competitively bind to human thyroid hormone transport protein transthyretin (TTR)^61^, which transports maternal thyroid hormones into the fetal circulation^62^. It was found that PFHxS had the highest binding potency, followed by PFOA. This might be one possible explanation of our negative correlations between maternal PFHxS and PFOA with fetal thyroid hormones, although only maternal PFOA still showed negative correlation with fetal FT3 after adjustment for covariates. And vice versa, the higher levels of maternal thyroid hormones might also inhibit the placental transfer of PFASs by competitive binding to PFAS transporter proteins, which could be a possible explanation of the negative correlations between maternal FT3 and T3 with most fetal PFASs found in our study. However, there is no such result reported before and the passive transport, instead of positive transport by transporter proteins, is considered as the major transporting manner for small molecules. Moreover, the variations in the levels of maternal thyroid hormones were smaller than that in the concentrations of maternal and fetal PFASs (coefficient of variation, CV: 17.60% for maternal FT3; 21.32% for maternal T3; 46.86–117.21% for PFASs), indicating the limited effect of maternal FT3 and T3 imposed on the placental transfer of PFASs by competitive protein binding. More investigations should be done to validate and explore the reason for this outcome.

As a result of complex environment, multiple exposure pathways and combined toxic effects made by other harmful pollutants, human exposure studies are always inconsistent with animal exposure experiments. Due to the discrepancy between different regions, race, community development degrees, industrial pollution degrees, sampling date, sex, age, and living habits of the participants and so on, the associations between PFASs and thyroid hormone levels are conflicting in different populations. The main strength of this study was the provided data for both prenatal exposure of PFASs and their potential associations with thyroid hormones in Beijing pregnant women. It was a first study to date to examine PFASs and thyroid disruption in pregnant population in the mainland of China. Moreover, it was also the first time to investigate the association between maternal thyroid hormones and fetal PFASs and several interesting negative correlations were found. Our study has several differences compared with previous researches. First, the maternal and fetal sera were collected around delivery, at which time the associations between PFASs and thyroid hormones turned to be complicated with the increasing autonomy of the fetal thyroid axis and the transient stress response happened at birth. However, sampling both maternal and cord sera around delivery could provide more reliable maternal-fetal correlations and placental transfer ratios of major PFASs. Second, we didn’t analysis the level of longer chain PFASs, such as perfluorotridecanoic acid (PFTrDA), which has been reported to have negative relationships with fetal T3 and T4^40^. And in one study of general population in Korea, PFTrDA was found negatively correlated with T4 and positively correlated with TSH in females^31^. For further investigations, besides PFASs, the combined effect on thyroid hormones homeostasis with other endocrine disrupting pollutants should be taken into consideration in future research^30^,^63^,^64^,^65^.

In conclusion, PFASs and precursors were detected in paired maternal and cord sera in Beijing pregnant women with significant maternal-fetal correlations and different placental transfer ratios. Maternal TSH was negatively correlated with most maternal PFASs while maternal T3, FT3 showed negative correlation with most fetal PFASs. Our results suggest prenatal exposure of fetus to PFASs and potential associations of PFASs and thyroid hormone homeostasis in humans.

## Method

### Serum Samples

157 pairs of maternal and cord serum samples were collected from women and their neonates in 2013. From January to March, volunteers of the pregnant women were randomly recruited. For the aim to investigate the prenatal exposure in Beijing general population and to exclude other influencing factors, volunteers were required as healthy local resident women who gave birth to a single live-born child without congenital anomalies. These women agreed to participate in the present study at Haidian Maternal & Child Health Hospital in Beijing, capital city of China. Participants were fully informed of the nature and purpose of the study and signed consent forms before participation in the study. The study protocol was reviewed and approved by the ethic committees of China National Center for Food Safety Risk Assessment and carried out in accordance with the approved guidelines. All the participants are healthy pregnant women having no medical histories of thyroid diseases or any other organic diseases. For each participant, information such as maternal age, BMI, maternal previous live births, neonatal sex, neonatal type of delivery, gestational weeks, neonate’s birth weight and birth length was collected from medical records since 13–16 weeks of pregnancy. Information of maternal monthly income was collected from self-questionnaire.

Cord blood samples were collected immediately after delivery, while maternal blood samples were collected within one or two days before delivery. The blood samples were centrifuged at 9384 × g for 15 min immediately after collection in hospital. Then the sera were transferred to prescreened 3 mL polypropylene containers and stored at −80 °C. Frozen samples were transported on dry ice to the laboratory at China National Center for Food Safety Risk Assessment for analysis.

### Chemicals

A list of all native and labeled standards of eight PFASs and fifteen PFAS precursors: PFHxA, PFOA, PFNA, PFDA, PFUnA, PFDoA, PFHxS, PFOS, three fluorotelomer sulfonates (FTSs), three perfluoroalkyl unsaturated carboxylates (FTUCAs), two perfluorooctane sulfonamides (PFOSAs), two Perfluorooctanesulfonamidoacetates (PFOSAAs), three perfluorophosphinates (PFPiAs) and two polyfluoroalkyl phosphate diesters (diPAPs) used in this study is provided in the Supplementary Information, p. S2. All solvents and reagents were of the highest commercial purity.

### Sample preparation

The serum samples were extracted using a modified version of the ion-pair extraction method developed by Hansen *et al.*^66^. A serum sample (0.5 mL) was mixed with internal standard solution (PFASs, 1 ng; precursors, 100 pg), 0.5 M tetra-n-butylammoniumhydrogen sulfate (TBA) solution (1 mL, adjusted to pH 10 with 2 mM sodium hydroxide solution) and 0.25 M sodium carbonate buffer (2 mL) in a 15 mL polypropylene tube. Methyl-tertbutyl ether (MTBE) (5 mL) was added to the solution for extraction. The organic and aqueous layers were separated by centrifugation. The aqueous mixture was rinsed twice with MTBE. All rinses were combined in a second polypropylene tube and evaporated at ambient temperature under nitrogen gas flow, and then reconstituted in 0.25 mL of methanol/water (1:1). The supernatant was filtered through a 0.2 μm nylon filter before analysis.

### Instrumental analysis

For the analysis of PFAS precursors, analytes were separated and quantified using an ultra-performance liquid chromatography system coupled to a triple quadrupole MS system (ACQUITY UPLC-Xevo TQ-S, Waters, USA). 2.1 × 50 mm BEH C_18_ column (1.7 μm; Waters, USA) was used in all instrumental analyses. A gradient of 2 mM aqueous ammonium acetate solution and methanol were used as mobile phases at a flow rate of 0.4 mL/min. The triple-quadrupole mass spectrometer was operated in the negative electrospray mode with multiple-reaction-monitoring (MRM). Detailed chromatographic gradients, instrumental conditions, and MRM mass transitions of PFAS precursors are provided in the Supplementary Information, p. S2 and Table S4 and Table S5. For the instrumental analysis of PFASs, the method has been described in detail elsewhere^6^,^50^.

### Quantification and quality assurance

Quantification was performed using an internal standard approach. Analytes without an isotope labeled standard were quantified using the internal standard with the closest retention time (see Supplementary Information, Table S4). Procedural blank analysis was conducted using Milli-Q water for each batch of samples. The limits of detection (LOD) and limits of quantification (LOQ) were defined as the concentrations producing a signal-to-noise (S/N) ratio equal to or greater than 3 and 10, respectively. For each precursor a six point calibration curve was made, ranging from 0.02–2.00 ng/mL. Calibration curves were linear over the concentration range with r values greater than 0.99 for all compounds. The recovery test was conducted by analyzing blank calf serum. Analyte recoveries were ranged from 41% to 128% (see Supplementary Information, Table S6). The reported concentrations in the human serum samples were not corrected for recovery. Details of quality control can be found in Supplementary Information p. S3. The LODs for PFASs were provided in the Supplementary Information, Table S7.

### Assessment of thyroid hormones

Serum concentrations of maternal and cord thyroid hormones, namely FT3, FT4, T3, T4 and TSH, were measured with chemo-luminescence immunoassay technology and an automated cobas e411 immunoassay analyzer (Roche Diagnostics, USA). Determinations were conducted following the manufacturer’s instructions. Assay ranges were 0.6–50 pmol/L, 1.3–100 pmol/L, 0.3–10 nmol/L, 5.40–320 nmol/L and 0.005–100 μIU/mL for FT3, FT4, T3, T4 and TSH, respectively. The intra-assay coefficients of variation (CVs) of these measures were all < 4% and the inter-assay CVs were all < 5%.

### Data analysis

For all statistical tests, undetectable PFAS concentration was accounted as a value equal to the LOD divided by the square root of 2^4^,^67^. The concentrations of PFDoA, 6:2 FTS, NMeFOSAA and T3 in both maternal and cord serum were non-normally distributed even after logarithmically transformation. For other PFASs and thyroid hormones, however, all the concentrations were normally distributed after log-transformed. As a result, Spearman and Pearson tests were used seperately to test the maternal-fetal correlations among the target analytes. Spearman partial correlation analysis was performed among the concentrations of thyroid hormones and PFASs in maternal and fetal serum with and without adjustment for influential covariates. Four women with abnormal thyroid hormones levels were excluded in correlation test. Covariates considered were maternal age, prepragnant BMI, gestation weeks, previous live births, delivery type, maternal income, fetal sex, birth length and birth weight of neonates. The covariates adjusted in the final partial correlation analysis were selected from a multivariate analysis (canonical correlation analysis, CCA) with *P* < 0.1 as a criterion. These covariates included maternal age, maternal prepregnancy BMI, maternal monthly income, and neonatal type of delivery for correlations between maternal PFASs and thyroid hormones and maternal prepregnancy BMI, maternal monthly income, maternal previous live births and neonatal type of delivery for correlations between fetal PFASs and thyroid hormones. Maternal prepregnancy BMI, maternal monthly income, neonatal birth length and neonatal type of delivery were covariates for correlations between maternal PFASs and fetal thyroid hormones, while maternal age, maternal prepregnancy BMI, maternal monthly income, maternal previous live births and neonatal type of delivery were covariates for correlations between fetal PFASs and maternal thyroid hormones (Table S12). All the statistical analyses were performed using the software of R 3.1.1. A *P*-value of 0.05 (2-tailed) was chosen as the criterion for statistical significance in all final analyses. The Spearman partial correlations with adjustment for covariates based on literature (Table S12) were also test and were presented in the Supplementary Information, Table S8-S11.

## Additional Information

**How to cite this article**: Yang, L. *et al.* Placental Transfer of Perfluoroalkyl Substances and Associations with Thyroid Hormones: Beijing Prenatal Exposure Study. *Sci. Rep.* **6**, 21699; doi: 10.1038/srep21699 (2016).

## Supplementary Material

Supplementary Information

## Figures and Tables

**Table 1:** Characteristics of the pregnant women (n = 157) and their neonates (n = 157). SD, standard deviation; n, number; BMI, body mass index; CNY, Chinese Yuan.

Characteristic	Mean ± SD or*n* (%)
Maternal age at enrollment (years)	29.8 ± 2.9
Maternal weight (kg)	55.5 ± 6.9
Maternal height (cm)	162.1 ± 4.4
Maternal prepregnancy BMI (kg/m^2^)	21.1 ± 2.4
Maternal previous live births	
0 (primiparous)	143 (91.1)
≥1 (multiparous)	14 (8.9)
Maternal monthly income (CNY, ¥)	
< 4000	43 (27.4)
≥4000 to <8000	87 (55.4)
≥8000	27 (17.2)
Neonatal sex	
Female	70 (44.6)
Male	87 (55.4)
Neonatal type of delivery	
Normal vaginal	74 (47.1)
Cesarean section	83 (52.9)
Neonatal gestational weeks at birth	39.8 ± 1.5
Neonatal birth weight (g)	3447.0 ± 420.4
Neonatal birth length (cm)	50.5 ± 1.1

**Table 2:** Serum concentrations of PFASs and PFAS precursors, associations and ratios between matched maternal and cord serum.

	PFHxS (ng/mL)	PFOS (ng/mL)	PFOA (ng/mL)	PFNA (ng/mL)	PFDA (ng/mL)	PFUnA (ng/mL)	PFDoA (ng/mL)	6:2 FTS (pg/mL)	NMeFOSAA (pg/mL)
Maternal serum^ab^									
Percent > LOD	100%	100%	100%	100%	100%	99%	68%	82%	74%
Mean ± SD	0.63 ± 0.47	5.08 ± 3.26	1.95 ± 1.09	0.52 ± 0.27	0.45 ± 0.28	0.45 ± 0.24	0.046 ± 0.026	48.38 ± 40.94	4.77 ± 6.68
Median	0.50	4.41	1.64	0.46	0.37	0.40	0.041	41.44	3.40
GM	0.53	4.23	1.74	0.46	037	0.38	0.040	26.04	2.06
Range	0.12–4.22	0.73–19.96	0.73–8.11	0.13–1.69	0.038–2.07	<LOD-1.27	<LOD-0.15	<LOD-203.00	<LOD-46.79
Cord serum^ab^									
Percent > LOD	100%	100%	100%	100%	100%	96%	33%	90%	66%
Mean ± SD	0.26 ± 0.30	1.52 ± 1.01	1.32 ± 0.69	0.23 ± 0.12	0.13 ± 0.09	0.14 ± 0.10	0.028 ± 0.013	33.54 ± 38.52	1.58 ± 2.36
Median	0.18	1.18	1.15	0.20	0.10	0.12	0.021	24.68	0.67
GM	0.19	1.27	1.18	0.21	0.10	0.11	0.026	19.98	0.76
Range	0.014–3.17	0.24–6.93	0.40–5.06	0.05–0.80	0.024–0.61	<LOD-0.75	<LOD-0.082	<LOD-252.12	<LOD-16.00
Maternal-fetal correlation^c^	r = 0.684 *P* < 0.001	r = 0.626 *P* < 0.001	r = 0.806 *P* < 0.001	r = 0.704 *P* < 0.001	r = 0.652 *P* < 0.001	r = 0.630 *P* < 0.001	r = 0.520 *P* < 0.001	r = 0.336 *P* < 0.001	r = 0.399 *P* < 0.001
Maternal and cord serum concentration ratio^d^									
Mean CS ± SD:MS	0.43 ± 0.34:1	0.36 ± 0.35:1	0.71 ± 0.22:1	0.49 ± 0.29:1	0.35 ± 0.44:1	0.36 ± 0.42:1	0.61 ± 0.34:1	3.08 ± 4.34:1	7.79 ± 11.14:1
Median CS:MS	0.35:1	0.29:1	0.65:1	0.43:1	0.25:1	0.27:1	0.51:1	1.66:1	3.83:1

LOD, limit of detection; SD, standard deviation; GM, geometric mean; CS, Cord Serum; MS, Maternal Serum. ^a^Undetectable concentration was accounted as a value equal to the LOD divided by the square root of 2 and values below the LOQ were used unaltered. ^b^Data of other analyates were not reported due to the low frequency of detection in the samples (<30% in both maternal and cord serum samples). ^c^Since concentrations of PFHxS, PFOS, PFOA, PFNA, PFDA and PFUnA were normally distributed after log-transformed, Pearson correlation test was used to test the log-transformed concentrations for possible correlations. Spearman correlation test was used to test the correlations of non-normally distributed PFDoA, 6:2 FTS and NMeFOSAA. ^d^For each compound, samples having undetectable concentration in both maternal and cord serum were excluded.

**Table 3:** Serum concentrations of thyroid hormones and their concentration associations between matched maternal and cord serum.

	FT3 (pmol/L)	FT4 (pmol/L)	T3 (nmol/L)	T4 (nmol/L)	TSH (μIU/mL)
Maternal serum					
*n*	123	156	155	157	157
Mean ± SD	3.79 ± 0.67	11.98 ± 2.03	2.57 ± 0.55	145.60 ± 35.82	3.32 ± 1.95
Median	3.82	11.89	2.54	140.00	3.03
GM	3.73	11.82	2.50	141.53	2.84
Range	1.76–5.41	7.45–17.49	0.77–3.86	71.42–288.30	0.26–12.77
Cord serum					
*n*	146	157	156	157	157
Mean ± SD	2.03 ± 0.52	15.94 ± 2.22	0.97 ± 0.36	129.52 ± 25.68	11.97 ± 8.66
Median	2.01	15.88	0.90	129.70	9.44
GM	1.98	15.78	0.93	126.98	9.86
Range	1.11–4.45	9.44–21.26	0.56–3.16	69.52–232.20	1.26–57.01
Maternal–fetal correlation^ab^	r = −0.139	r = 0.083	r = −0.026	r = 0.260	r = 0.001
	*P* = 0.142	*P* = 0.303	*P* = 0.751	*P* = 0.001	*P* = 0.993

LOD, limit of detection; SD, standard deviation; GM, geometric mean. ^a^Exclude cases pairwise. ^b^Since concentrations of FT3, FT4, T4 and TSH were normally distributed after log-transformed, Pearson correlation test was used to test the log-transformed concentrations for possible correlations. Spearman correlation test was used to test the correlations of non-normally distributed T3.

**Table 4:** Correlations between maternal PFASs and maternal thyroid hormones.

Maternal PFASs	Maternal FT3		Maternal FT4		Maternal T3		Maternal T4		Maternal TSH	
	Not-adjusted	Adjusted^a^	Not-adjusted	Adjusted^a^	Not-adjusted	Adjusted^a^	Not-adjusted	Adjusted^a^	Not-adjusted	Adjusted^a^
PFOA	0.054	0.024	−0.013*	0.000	0.114	0.102	0.083	0.062	−0.162**	−0.124
PFNA	−0.048**	−0.063	−0.085**	−0.072	−0.021*	−0.018	0.010*	−0.006	−0.180**	−0.170*
PFDA	−0.060**	−0.087	−0.077**	−0.086	−0.075**	−0.079	0.019*	−0.010	−0.218**	−0.216**
PFUnA	−0.119**	−0.121	−0.061**	−0.062	−0.109**	−0.097	0.044*	0.030	−0.184**	−0.202*
PFDoA	−0.262**	−0.268**	−0.120**	−0.160*	−0.292**	−0.301**	−0.114**	−0.160*	−0.197**	−0.231**
PFHxS	0.119	0.124	0.047	0.038	0.072	0.084	0.108	0.084	−0.149*	−0.154
PFOS	0.012*	0.025	−0.041**	−0.057	−0.019*	0.008	0.050*	0.021	−0.236**	−0.261**
6:2 FTS	0.187	0.160*	0.139	0.164*	0.216*	0.205*	0.183	0.172*	−0.067	−0.013
NMeFOSAA	0.152	0.163*	0.037	0.081	−0.039*	−0.028	0.024	0.072	−0.009*	−0.046

Spearman correlation tests were used among the concentrations of PFASs and thyroid hormones with- and without adjustment influential covariates, which were selected from multivariate models. Four women with abnormal thyroid hormones levels were excluded. Units in pg/mL for 6:2 FTS and NMeFOSAA, ng/mL for other PFASs, pmol/mL for FT3 and FT4, nmol/mL for T3 and T4, μIU/mL for TSH. ^a^Adjusted for maternal age, maternal prepregnancy BMI, maternal monthly income, and neonatal type of delivery. * *P* < 0.05, ** *P* < 0.01.

**Table 5:** Correlations between fetal PFASs and maternal thyroid hormones.

	Maternal FT3		Maternal FT4		Maternal T3		Maternal T4		Maternal TSH	
Fetal PFASs	Not-adjusted	Adjusted^a^	Not-adjusted	Adjusted^a^	Not-adjusted	Adjusted^a^	Not-adjusted	Adjusted^a^	Not-adjuste	d Adjusted^a^
PFOA	−0.164**	−0.169*	−0.053**	−0.089	−0.072**	−0.069	−0.040**	−0.069	−0.073**	−0.065
PFNA	−0.260**	−0.243**	−0.095**	−0.101	−0.201**	−0.165*	−0.099**	−0.103	−0.039**	−0.045
PFDA	−0.264**	−0.264**	−0.091**	−0.126	−0.249**	−0.229**	−0.101**	−0.132	−0.124**	−0.135
PFUnA	−0.293**	−0.293**	−0.037**	−0.072	−0.246**	−0.213**	−0.028**	−0.045	−0.130**	−0.155
PFDoA	−0.268**	−0.255**	−0.090**	−0.097	−0.244**	−0.224**	−0.125**	−0.134	0.047*	0.061
PFHxS	−0.185**	−0.192*	0.045*	−0.006	−0.176**	−0.172*	−0.010	−0.034	−0.028**	−0,066
PFOS	−0.249**	−0.215**	−0.004**	−0.059	−0.248**	−0.203*	−0.071**	−0.108	−0.156*	−0.173*
6:2 FTS	−0.096	−0.122	−0.002	−0.001	−0.080	−0.076	−0.048	−0.056	0.117	0.127
NMeFOSAA	0.034*	0.060	−0.014*	−0.025	−0.046*	−0.025	−0.024	−0.023	0.001**	−0.007

Spearman correlation tests were used among the concentrations of PFASs and thyroid hormones with- and without adjustment influential covariates, which were selected from multivariate models. Four women with abnormal thyroid hormones levels were excluded. Units in pg/mL for 6:2 FTS and NMeFOSAA, ng/mL for other PFASs, pmol/mL for FT3 and FT4, nmol/mL for T3 and T4, μIU/mL for TSH. ^a^Adjusted for maternal age, maternal prepregnancy BMI, maternal monthly income, maternal previous live births and neonatal type of delivery. *P < 0.05, **P < 0.01.

**Table 6:** Summary of the results from different pregnant women populations.

Country (year^a^)	Trimester	Mean age (n)	PFASs median levels	Major associations (M: maternal; C: cord)	Reference
Canada (2005–2006)	2nd trimester	31. 6 years (96 cases) 31.0 years (175 controls)	Case/Control levels (nmol/L)PFOA: 3.94/3.62 PFOS: 15.50/16.43PFHxS: 2.47/2.35	No correlations	Chan *et al.*^39^
South Korea (2008–2009)	Most in 3rd trimester	32 years (44)	Maternal/Fetal levels (ng/mL)PFOA: 1.46/1.15 PFOS: 2.93/1.26 PFUnA: 0.60/–PFNA: 0.44/0.45 PFHxS: 0.55/0.34 PFTrDA: 0.24/0.47PFDA: 0.31/0.19 PFHpS: 0.09/0.06	M-PFOS & C-T3 (negative)M-PFTrDA & C-T3, C-T4 (positive)	Kim *et al.*^40^
Norway (2003–2004)	2nd trimester	30 years (903)	Maternal levels (ng/mL)PFOA: 2.15 PFOS: 12.81 PFUnA: 0.22PFNA: 0.39 PFHxS: 0.60PFDA: 0.09 PFHpS: 0.13	M- PFOS & M-TSH (positive)	Wang *et al.*^41^
Taiwan, China (2000–2001)	3rd trimester	28.8 years (285)	Maternal levels (ng/mL)PFOA: 2.39 PFOS: 12.73 PFUnA: 3.26PFNA: 1.51 PFHxS: 0.81 PFDoA: 0.36PFDA: 0.46	M-PFNA, M-PFUnA, M-PFDoA& M-FT4, M-T4 (negative)& C-T3, T4 (negative)M-PFDA & M-T3 (positive)& C-T3 (negative)M-PFHxS & M-TSH (positve)	Wang *et al.*^42^
Norway (2007–2009)	2nd trimester	31 years (378)	Maternal levels (ng/mL)PFOA: 1.53 PFOS: 8.03 PFUnA: 0.26PFNA: 0.56 PFHxS: 0.44PFDA: 0.23 PFHpS: 0.10	Women within the highest PFOS quartile (11.1-35.9 ng/mL) has higher TSH	Berg *et al.*^44^
Beijing, China (2013)	1–2 day before delivery	29.8 years (157)	Maternal/Fetal levels (ng/mL)PFOA: 1.64/1.15 PFOS: 4.41/1.18 PFUnA: 0.40/0.12PFNA: 0.46/0.20 PFHxS: 0.50/0.18 PFDoA: 0.041/0.021PFDA: 0.37/0.10	M-PFASs & M-TSH (negative) C-PFASs & M-T3, M-FT3 (negative)	This study

M: maternal; C: cord; n, number. ^*a*^Sampling year.
