# Supplementary material for: Placental Transfer of Perfluoroalkyl Substances and Associations with Thyroid Hormones: Beijing Prenatal Exposure Study
Source: Sci Rep. 2016 Feb 22;6:21699. doi: 10.1038/srep21699 (PMC4762009; doi:10.1038/srep21699)
Supplement: Supplementary Information [file srep21699-s1.doc]

**Supplementary Information**

**Placental Transfer of Perfluoroalkyl Substances and Associations with Thyroid Hormones: Beijing Prenatal Exposure Study**

Lin Yang^1,3^, Jingguang Li^1^, Jianqiang Lai^2^, Hemi Luan^3^, Zongwei Cai^3^, Yibaina Wang^1^, Yunfeng Zhao^1^, Yongning Wu^1^

^1^Key Laboratory of Food Safety Risk Assessment, Ministry of Health and China National Center for Food Safety Risk Assessment, No. 7, Panjiayuannanli, 100021, Beijing, China.

^2^National Institute of Nutrition and Health, Chinese Centre for Disease Control and Prevention

^3^State Key Laboratory of Environmental and Biological Analysis, Department of Chemistry, Hong Kong Baptist University, Kowloon Tong, Hong Kong SAR, China

*Corresponding author: Jingguang Li and Jianqiang Lai; E-mail: [Lijg@cfsa.net.cn](mailto:Lijg@cfsa.net.cn), jq_lai@126.com.

| **Table of contents** | **page** |  |
| --- | --- | --- |
| **Experimental** | S2 |  |
| Chemicals | S2 |  |
| Instrumental analysis | S2 |  |
| Details of quality control | S3 |  |
| **References** | S4 |  |
| **Tables** |  |  |
| **Table S1.** Correlations among maternal PFASs. | S5 | |
| **Table S2.** Correlations between fetal PFASs and fetal thyroid hormones. | S6 | |
| **Table S3.** Correlations between maternal PFASs and fetal thyroid hormones | S7 | |
| **Table S4.** Multiple reaction monitoring (MRM) transitions and masss pectrometry parameters for PFAS precursors. | S8 | |
| **Table S5.** Multiple reaction monitoring (MRM) transitions and mass spectrometry parameters for internal standards. | S9 |  |
| **Table S6.** Limits of detection (LODs), limits of quantification (LOQs), and matrix recoveries for PFAS precursors. | S10 |  |
| **Table S7.** Limits of detection (LODs) for PFASs | S11 |  |
| **Table S8.** Correlations between maternal PFASs and maternal thyroid hormones adjusted by literature covariates. | S12 |  |
| **Table S9.** Correlations between fetal PFASs and fetal thyroid hormones adjusted by literature covariates. | S13 |  |
| **Table S10.** Correlations between maternal PFASs and fetal thyroid hormones adjusted by literature covariates. | S14 |  |
| **Table S11.** Correlations between fetal PFASs and maternal thyroid hormones adjusted by literature covariates. | S15 |  |
| **Table S12.** Comparation of covariates selected from multivariate analysis and literatures. | S16 |  |
| **Figure** |  |  |
| **Fig. S1.** Composition profiles of major PFASs in maternal and cord serum. | S17 |  |

**Experimental**

**Chemicals.** perfluorohexanesulfonate (PFHxS), perfluorooctanesulfonate (PFOS), Perfluorohexanoic acid (PFHxA), perfluorooctanoic acid (PFOA), perfluorononanoic acid (PFNA), perfluorodecanoic acid (PFDA), perfluoroundecanoic acid (PFUnA), perfluorododecanoic acid (PFDoA), 4:2, 6:2, and 8:2 fluorotelomer sulfonates (4:2, 6:2, 8:2 FTS), 2H-Perfluro-2-octenoic acid (FHUEA), 2H-Perfluro-2-decenoic acid (FOUEA), 2H-Perfluro-2-dodecenoic acid (FDUEA), perfluorooctane sulfonamide (PFOSA), *N*-ethyl perfluorooctane sulfonamide (NEtFOSA), *N*-methyl perfluorooctanesulfonamidoacetate (NMeFOSAA), *N*-ethyl perfluorooctanesulfonamidoacetate (NEtFOSAA), C6/C6, C6/C8 and C8/C8 bis(perfluorohexyl)phosphinate (C6/C6, C6/C8 and C8/C8 PFPiA), 6:2 and 8:2 polyfluoroalkyl phosphate diesters (6:2 and 8:2 diPAPs) were supplied by Wellington Laboratories (Guelph,Canada). Mass-labeled internal standards were also from Wellington Laboratories and they included: ^13^C_2_-PFHxA, ^13^C_4_-PFOA, ^13^C_5_-PFNA, ^13^C_2_-PFDA, ^13^C_2_-PFUnA, ^13^C_2_-PFDoA, ^18^O_2_-PFHxS, ^13^C_4_-PFOS, ^13^C_2_-6:2 FTS, ^13^C_2_-FHUEA, ^13^C_2_-FOUEA, ^13^C_2_-FDUEA, ^13^C_8_-PFOSA, d_5_-NEtFOSA, d_3_-NMeFOSAA, d_5_-NEtFOSAA, ^13^C_4_-6:2 diPAP, ^13^C_4_-8:2 diPAP.

Methanol of high performance liquid chromatography (HPLC) grade was purchased from J&K Scientific Ltd. (Beijing, China). Milli-Q water was used throughout the study. Tetra-n-butylammoniumhydrogen sulfate (TBA) was purchased from J&K Scientific Ltd. (Beijing, China), and methyl-tert-butyl ether (MTBE) was from Sigma-Aldrich (St. Louis, USA). HPLC grade ammonium acetate and formic acid were from Dikma Technologies Inc (Lake forest, USA). Analytical grade sodium carbonate and sodium hydroxide were purchased from Beijing Chemical Works (Beijing, China).

**Instrumental analysis.** For the analysis of PFAS precursors, the samples were injected as 10 μL injections and analyzed by the following gradient method at 0.4 mL/min using HPLC grade methanol and 2 mM ammonium acetate aqueous solution: the initial solvent composition at t = 0 min was 60 : 40 acetate aqueous solution : methanol and held for 0.5 min, then changed to 10 : 90 over a period of 11.5 min, at t = 12.0 min, before returning to the initial composition of 60 : 40 acetate aqueous solution : methanol at t = 14.0 min. The column was allowed to rebalance for 1.5 min, for a total run time of 15.5 min. The column temperature was set at 40 °C. The mass spectrometer operated in negative ion electrospray ionization (ESI^−^) mode. The capillary voltage was set at 3.0 kV. The source and desolvation temperatures were 120 °C and 400 °C, respectively. The desolvation and cone gas flow (nitrogen) were set at 800 L/h and 150 L/h, respectively. Compound-specifically optimized cone voltages and collision energies were listed in Table S4. For the analysis of PFASs, the analytical method and the mass transition for each compound have been described elsewhere in detail^1,2^. The LODs for PFASs were provided in Table S7.

**Details of quality control.** Two blank samples (Milli-Q water) were extracted with each batch of 20 samples and water was injected before the sample sequence to check instrumental background. No contamination of PFASs and precursors was found above the limit of detection (LOD) in solution and reagents used in this analysis. Mixture of standard was injected with every 10 injections to check the instrumental performance. Acetonitrile and water were injected in sequence with every 4 or 5 injections to clean system accumulated 6:2 diPAP and 8:2 diPAP residuals. The containers like tubes for blood collection were extracted with methanol and contained no PFASs and precursors above the LOD. The analysis system and the filters used in sample preparation were all washed by water and methanol before analyzing. Quantification was performed using an internal standard approach. For C6/C6, C6/C8 and C8/C8 PFPiA, there was no internal standard available. Concentrations of the three analytes in serum were planned to be quantified by standard addition reported by Mabury group^3,4^. However, none of them has been detected in the serum samples. The stability of PFAS precursors was tested. No obviously degradation was observed during sample storage at −80 °C (retested with an interval of 4 months) and multigelation (between −80 °C and room temperature for 3 times). Furthermore, the PFASs levels measured in our study were successfully validated by analyzing human serum SRM1957 and SRM1958 purchased from National Institute of Standards and Technology.

**References**

1. Liu, J., Li, J., Luan, Y., Zhao, Y., Wu, Y. Geographical distribution of perfluorinated compounds in human blood from Liaoning province, China. *Environ. Sci. Technol.* **43**, 4044-4048 (2009).

2. Liu, J. *et al.* Comparison on gestation and lactation exposure of perfluorinated compounds for newborns. *Environ. Int.* **37**, 1206-1212 (2011).

3. D'Eon, J. C., Crozier, P. W., Furdui, V. I., Reiner, E. J., Libelo, E. L., Mabury, S. A. Observation of a commercial fluorinated material, the polyfluoroalkyl phosphoric acid diesters, in human sera, wastewater treatment plant sludge, and paper fibers. *Environ. Sci. Technol.* **43**, 4589-4594 (2009).

4. Lee, H., Mabury, S. A. A pilot survey of legacy and current commercial fluorinated chemicals in human sera from United States donors in 2009. *Environ. Sci. Technol.* **45**, 8067-8074 (2011).

| **Correlaitons** | **PFHxS** | **PFOS** | **PFOA** | **PFNA** | **PFDA** | **PFUnA** | **PFDoA***^a^* | **6:2 FTS***^a^* | **NMeFOSAA***^a^* |
| --- | --- | --- | --- | --- | --- | --- | --- | --- | --- |
| PFHxS | 1 |  |  |  |  |  |  |  |  |
| PFOS | 0.501** | 1 |  |  |  |  |  |  |  |
| PFOA | 0.274** | 0.289** | 1 |  |  |  |  |  |  |
| PFNA | 0.298** | 0.684** | 0.559** | 1 |  |  |  |  |  |
| PFDA | 0.193* | 0.715** | 0.436** | 0.876** | 1 |  |  |  |  |
| PFUnA | 0.185* | 0.696** | 0.374** | 0.832** | 0.932** | 1 |  |  |  |
| PFDoA*^a^* | 0.067 | 0.409** | 0.056 | 0.413** | 0.492** | 0.557** | 1 |  |  |
| 6:2 FTS*^a^* | 0.070 | 0.100 | 0.254** | 0.157* | 0.130 | 0.139 | -0.143 | 1 |  |
| NMeFOSAA*^a^* | 0.171* | 0.241** | 0.058 | 0.148 | 0.121 | 0.174* | 0.081 | 0.031 | 1 |

**Table S1. Correlations among maternal PFASs.** *^a^*Spearman tests were used among the non-normal distributed concentrations of PFDoA, 6:2 FTS and NMeFOSAA, while others were log-transferred and used Pearson test. Units in pg/mL for 6:2 FTS and NMeFOSAA, in ng/mL for other PFASs. * *P* < 0.05, ** *P* < 0.01.

| **Fetal PFASs** | **Fetal FT3**  **Not-adjusted Adjusted^a^** | | **Fetal FT4**  **Not-adjusted Adjusted^a^** | | **Fetal T3**  **Not-adjusted Adjusted^a^** | | **Fetal T4**  **Not-adjusted Adjusted^a^** | | **Fetal TSH**  **Not-adjusted Adjusted^a^** | |
| --- | --- | --- | --- | --- | --- | --- | --- | --- | --- | --- |
| PFOA | -0.111 | -0.105 | -0.076* | -0.045 | -0.049 | -0.055 | 0.090 | 0.107 | -0.027 | -0.057 |
| PFNA | 0.059 | 0.094 | -0.050 | -0.023 | 0.083 | 0.118 | 0.134 | 0.147 | -0.074 | -0.105 |
| PFDA | 0.107 | 0.154 | -0.012 | 0.027 | 0.140 | 0.188* | 0.158 | 0.181* | -0.067 | -0.145 |
| PFUnA | 0.065 | 0.108 | -0.010 | 0.033 | 0.070 | 0.113 | 0.149 | 0.172* | -0.025 | -0.094 |
| PFDoA | 0.082 | 0.116 | -0.018 | 0.011 | 0.092 | 0.071 | 0.023 | 0.058 | 0.087 | 0.016 |
| PFHxS | 0.113 | 0.125 | -0.083 | -0.016 | 0.115 | 0.108 | 0.076 | 0.111 | -0.002 | -0.034 |
| PFOS | 0.137 | 0.191* | -0.005 | 0.053 | 0.115 | 0.170* | 0.136 | 0.172* | -0.033 | -0.119 |
| 6:2 FTS | -0.106** | -0.078 | -0.180** | -0.109 | -0.042* | -0.008 | -0.101 | -0.075 | 0.007 | -0.054 |
| NMeFOSAA | -0.068 | -0.076 | 0.021 | 0.023 | -0.101* | -0.104 | 0.008 | 0.004 | 0.071 | 0.097 |

**Table S2. Correlations between fetal PFASs and fetal thyroid hormones.** Spearman correlation tests were used among the concentrations of PFASs and thyroid hormones with- and without adjustment influential covariates, which were selected from multivariate models. Units in pg/mL for 6:2 FTS and NMeFOSAA, ng/mL for other PFASs, pmol/mL for FT3 and FT4, nmol/mL for T3 and T4, μIU/mL for TSH. ^a^Adjusted for maternal prepregnancy BMI, maternal monthly income, maternal previous live births and neonatal type of delivery. * *P* < 0.05, ** *P* < 0.01.

| **Maternal PFASs** | **Fetal FT3**  **Not-adjusted Adjusted^a^** | | **Fetal FT4**  **Not-adjusted Adjusted^a^** | | **Fetal T3**  **Not-adjusted Adjusted^a^** | | **Fetal T4**  **Not-adjusted Adjusted^a^** | | **Fetal TSH**  **Not-adjusted Adjusted^a^** | |
| --- | --- | --- | --- | --- | --- | --- | --- | --- | --- | --- |
| PFOA | -0.160* | -0.169* | -0.013 | -0.014 | -0.134* | -0.148 | -0.003 | -0.005 | -0.018 | -0.016 |
| PFNA | -0.008 | 0.008 | 0.077 | 0.127 | -0.029 | -0.009 | 0.053 | 0.065 | -0.012 | -0.044 |
| PFDA | 0.005 | 0.059 | 0.071 | 0.141 | -0.012 | 0.035 | 0.051 | 0.073 | 0.020 | -0.039 |
| PFUnA | 0.024 | 0.059 | 0.030 | 0.091 | 0.003 | 0.027 | 0.057 | 0.077 | 0.015 | -0.047 |
| PFDoA | 0.029 | 0.034 | 0.002 | 0.027 | 0.010 | 0.005 | 0.053 | 0.053 | 0.000 | -0.058 |
| PFHxS | -0.023** | -0.025 | -0.027* | 0.010 | -0.048** | -0.067 | -0.008* | -0.005 | 0.117** | 0.083 |
| PFOS | 0.054 | 0.088 | 0.036 | 0.120 | 0.007* | 0.040 | 0.055 | 0.093 | 0.081* | -0.008 |
| 6:2 FTS | 0.073 | 0.102 | -0.023 | 0.032 | 0.063 | 0.100 | -0.017 | 0.000 | 0.005 | -0.071 |
| NMeFOSAA | -0.077 | -0.123 | 0.057 | 0.025 | -0.019 | -0.066 | 0.127 | 0.107 | 0.051* | 0.076 |

**Table S3. Correlations between maternal PFASs and fetal thyroid hormones.** Spearman correlation tests were used among the concentrations of PFASs and thyroid hormones with- and without adjustment influential covariates, which were selected from multivariate models. Units in pg/mL for 6:2 FTS and NMeFOSAA, ng/mL for other PFASs, pmol/mL for FT3 and FT4, nmol/mL for T3 and T4, μIU/mL for TSH. ^a^Adjusted for maternal prepregnancy BMI, maternal monthly income, neonatal birth length and neonatal type of delivery. * *P* < 0.05, ** *P* < 0.01.

| **Analyte** | **Mass Transition** | **Cone Voltage (v)** | **Collision Energy (eV)** | **Internal Standard used** |
| --- | --- | --- | --- | --- |
| 4:2 FTS | 327>81  327>307 | 8  8 | 24  18 | ^13^C_2_-6:2 FTS |
| 6:2 FTS | 427>81  427>407 | 60  60 | 26  22 | ^13^C_2_-6:2 FTS |
| 8:2 FTS | 527>81  527>507 | 76  76 | 30  22 | ^13^C_2_-6:2 FTS |
| FHUEA | 357>293 | 8 | 14 | ^13^C_2_-FHUEA, |
| FOUEA | 457>393 | 20 | 22 | ^13^C_2_-FOUEA, |
| FDUEA | 557>493 | 6 | 24 | ^13^C_2_-FDUEA, |
| PFOSA | 498>78 | 76 | 26 | ^13^C_8_-PFOSA |
| NEtFOSA | 526>169 | 78 | 24 | d_5_-NEtFOSA |
| NMeFOSAA | 570>419 | 48 | 18 | d_3_-NMeFOSAA |
| NEtFOSAA | 584>419 | 50 | 22 | d_3_-NEtFOSAA |
| 6:6 PFPiA | 701>401 | 98 | 44 | --^a^ |
| 6:8 PFPiA | 801>401  801>501 | 82  82 | 52  50 | --^a^ |
| 8:8 PFPiA | 901>501 | 82 | 56 | --^a^ |
| 6:2 diPAP | 789>97  789>443 | 32  32 | 36  20 | ^13^C_4_-6:2 diPAP |
| 8:2 diPAP | 989>97  989 >543 | 70  70 | 36  20 | ^13^C_4_-8:2 diPAP |

**Table S4. Multiple reaction monitoring (MRM) transitions and mass spectrometry parameters for PFAS precursors.** ^a^C6/C6, C6/C8 and C8/C8 PFPiA have no internal standards available.

| **Analyte** | **Mass Transition** | **Cone Voltage (v)** | **Collision Energy (eV)** |
| --- | --- | --- | --- |
| ^13^C_2_-6:2 FTS | 429>81  429>409 | 44  44 | 34  22 |
| ^13^C_2_-FHUEA, | 359>294 | 10 | 12 |
| ^13^C_2_-FOUEA, | 459>394 | 10 | 14 |
| ^13^C_2_-FDUEA, | 559>494 | 18 | 22 |
| ^13^C_8_-PFOSA | 506>78 | 80 | 26 |
| d_5_-NEtFOSA | 531>169 | 72 | 28 |
| d_3_-NMeFOSAA | 573>419 | 34 | 18 |
| d_3_-NEtFOSAA | 589>419 | 52 | 18 |
| ^13^C_4_-6:2 diPAP | 793>97  793>445 | 64  64 | 32  18 |
| ^13^C_4_-8:2 diPAP | 993>97  993>545 | 78  78 | 32  22 |

**Table S5. Multiple reaction monitoring (MRM) transitions and mass spectrometry parameters for internal standards.**

| **Compounds** | **Mean Recovery (%), LOD and LOQ** | | | | |
| --- | --- | --- | --- | --- | --- |
|  | **low level^a^** | **med level^b^** | **high level^c^** | **LOD (pg/mL)** | **LOQ**  **(pg/mL)** |
| 4:2 FTS | 80±10 | 100±7 | 98±1 | 4 | 10 |
| 6:2 FTS | 88±9 | 97±7 | 98±3 | 3 | 10 |
| 8:2 FTS | 77±10 | 80±9 | 101±4 | 4 | 10 |
| FHUEA | 102±11 | 105±7 | 107±2 | 2 | 8 |
| FOUEA | 106±6 | 112±6 | 103±2 | 0.5 | 2 |
| FDUEA | 104±15 | 121±15 | 105±2 | 1 | 2 |
| PFOSA | 109±4 | 127±3 | 102±3 | 0.2 | 0.5 |
| NEtFOSA | 98±8 | 101±10 | 101±3 | 0.2 | 0.5 |
| NMeFOSAA | 70±6 | 78±4 | 103±4 | 0.3 | 1 |
| NEtFOSAA | 100±8 | 128±5 | 74±2 | 0.4 | 1 |
| 6:6 PFPiA | 78±12 | 112±10 | 92±2 | 1 | 3 |
| 6:8 PFPiA | 52±9 | 75±4 | 58±6 | 1 | 2 |
| 8:8 PFPiA | 41±10 | 53±5 | 45±8 | 0.2 | 0.5 |
| 6:2 diPAP | 100±12 | 109±10 | 102±8 | 1 | 2 |
| 8:2 diPAP | 106±6 | 112±2 | 100±5 | 0.5 | 1 |

**Table S6. Limits of detection (LODs), limits of quantification (LOQs), and matrix recoveries for PFAS precursors.** LOD, limit of detection; LOQ, limit of quantification. ^a^20 pg/mL for all precursors (n=5). ^b^100 pg/mL for all precursors (n=5). ^c^1 ng/mL for all precursors (n=5).

| **Compounds** |  |
| --- | --- |
|  | **LOD (ng/mL)** |
| PFHxS | 0.012 |
| PFOS | 0.021 |
| PFHxA | 0.009 |
| PFOA | 0.024 |
| PFNA | 0.013 |
| PFDA | 0.024 |
| PFUnA | 0.033 |
| PFDoA | 0.029 |

**Table S7. Limits of detection (LODs) for PFASs.** LOD, limit of detection.

| **Maternal PFAS** | **Maternal FT3**  **No Income Adjusted** | | **Maternal FT4**  **No Income Adjusted** | | **Maternal T3**  **No Income Adjusted** | | **Maternal T4**  **No Income Adjusted** | | **Maternal TSH**  **No Income Adjusted** | |
| --- | --- | --- | --- | --- | --- | --- | --- | --- | --- | --- |
| PFOA | 0.060 | 0.047 | -0.004 | -0.001 | 0.123 | 0.115 | 0.069 | 0.071 | -0.148 | -0.152 |
| PFNA | -0.050 | -0.048 | -0.062 | -0.055 | -0.021 | -0.008 | 0.006 | 0.009 | -0.163* | -0.174* |
| PFDA | -0.061 | -0.051 | -0.066 | -0.074 | -0.079 | -0.066 | 0.004 | -0.010 | -0.193* | -0.214** |
| PFUnA | -0.113 | -0.102 | -0.039 | -0.043 | -0.094 | -0.082 | 0.041 | 0.035 | -0.164* | -0.196* |
| PFDoA | -0.249** | -0.251** | -0.137 | -0.150 | -0.297** | -0.294** | -0.144 | -0.163* | -0.202* | -0.224** |
| PFHxS | 0.134 | 0.131 | -0.015 | 0.028 | 0.079 | 0.083 | 0.080 | 0.097 | -0.209** | -0.204* |
| PFOS | 0.040 | 0.027 | -0.046 | -0.050 | -0.004 | -0.008 | 0.034 | 0.021 | -0.251** | -0.255** |
| 6:2 FTS | 0.170* | 0.174* | 0.167* | 0.173* | 0.214** | 0.205* | 0.161* | 0.168* | -0.007 | -0.009 |
| NMeFOSAA | 0.159 | 0.154 | 0.071 | 0.082 | -0.029 | -0.030 | 0.055 | 0.065 | -0.050 | -0.058 |

**Table S8. Correlations between maternal PFASs and maternal thyroid hormones adjusted by literature covariates.** Spearman correlation tests were used among the concentrations of PFASs and thyroid hormones with adjustment influential covariates based on literature. Four women with abnormal thyroid hormones levels were excluded. Units in pg/mL for 6:2 FTS and NMeFOSAA, ng/mL for other PFASs, pmol/mL for FT3 and FT4, nmol/mL for T3 and T4, μIU/mL for TSH. Adjusted for maternal age, gestation weeks, maternal prepregnancy BMI, previous live births, with- or without maternal monthly income. * *P* < 0.05, ** *P* < 0.01.

| **Fetal PFAS** | **Fetal FT3**  **No GW Adjusted** | | **Fetal FT4**  **No GW Adjusted** | | **Fetal T3**  **No GW Adjusted** | | **Fetal T4**  **No GW Adjusted** | | **Fetal TSH**  **No GW Adjusted** | |
| --- | --- | --- | --- | --- | --- | --- | --- | --- | --- | --- |
| PFOA | -0.127 | -0.112 | -0.031 | -0.047 | -0.086 | -0.056 | 0.112 | 0.110 | -0.054 | -0.059 |
| PFNA | 0.071 | 0.075 | -0.025 | -0.030 | 0.082 | 0.087 | 0.152 | 0.148 | -0.090 | -0.099 |
| PFDA | 0.130 | 0.131 | 0.019 | 0.018 | 0.153 | 0.158* | 0.176* | 0.174* | -0.129 | -0.128 |
| PFUnA | 0.087 | 0.088 | 0.031 | 0.029 | 0.094 | 0.093 | 0.186* | 0.184* | -0.067 | -0.068 |
| PFDoA | 0.115 | 0.117 | 0.029 | 0.026 | 0.096 | 0.097 | 0.083 | 0.083 | 0.020 | 0.022 |
| PFHxS | 0.091 | 0.091 | -0.014 | -0.030 | 0.065 | 0.073 | 0.113 | 0.102 | -0.045 | -0.037 |
| PFOS | 0.181* | 0.190* | 0.074 | 0.068 | 0.142 | 0.148 | 0.183* | 0.172* | -0.115 | -0.108 |
| 6:2 FTS | -0.070 | -0.069 | -0.123 | -0.119 | 0.010 | 0.012 | -0.092 | -0.099 | -0.051 | -0.048 |
| NMeFOSAA | -0.079 | -0.079 | 0.021 | 0.029 | -0.110 | -0.111 | 0.018 | 0.020 | 0.093 | 0.100 |

**Table S9. Correlations between fetal PFASs and fetal thyroid hormones adjusted by literature covariates.** GW, gestation weeks. Spearman correlation tests were used among the concentrations of PFASs and thyroid hormones with adjustment influential covariates based on literature. Units in pg/mL for 6:2 FTS and NMeFOSAA, ng/mL for other PFASs, pmol/mL for FT3 and FT4, nmol/mL for T3 and T4, μIU/mL for TSH. Adjusted for maternal age, maternal prepregnancy BMI, neonatal sex, previous live births, neonatal type of delivery, with- or without gestation weeks. * *P* < 0.05, ** *P* < 0.01.

| **Maternal PFAS** | **Fetal FT3**  **No GW Adjusted** | | **Fetal FT4**  **No GW Adjusted** | | **Fetal T3**  **No GW Adjusted** | | **Fetal T4**  **No GW Adjusted** | | **Fetal TSH**  **No GW Adjusted** | |
| --- | --- | --- | --- | --- | --- | --- | --- | --- | --- | --- |
| PFOA | -0.178* | -0.180* | 0.004 | -0.001 | -0.163* | -0.154 | -0.003 | -0.006 | -0.027 | -0.022 |
| PFNA | -0.004 | -0.008 | 0.099 | 0.092 | -0.033 | -0.031 | 0.062 | 0.063 | -0.020 | -0.024 |
| PFDA | 0.028 | 0.027 | 0.098 | 0.094 | 0.005 | 0.006 | 0.064 | 0.063 | -0.013 | -0.013 |
| PFUnA | 0.037 | 0.041 | 0.062 | 0.065 | 0.021 | 0.020 | 0.082 | 0.083 | -0.012 | -0.011 |
| PFDoA | 0.033 | 0.036 | 0.021 | 0.025 | 0.018 | 0.016 | 0.069 | 0.070 | -0.017 | -0.017 |
| PFHxS | -0.042 | -0.049 | 0.053 | 0.049 | -0.090 | -0.092 | 0.033 | 0.028 | 0.070 | 0.078 |
| PFOS | 0.085 | 0.087 | 0.128 | 0.126 | 0.032 | 0.030 | 0.103 | 0.101 | -0.001 | 0.002 |
| 6:2 FTS | 0.101 | 0.105 | 0.006 | 0.013 | 0.101 | 0.106 | -0.016 | -0.014 | -0.074 | -0.077 |
| NMeFOSAA | -0.119 | -0.123 | 0.022 | 0.027 | -0.070 | -0.074 | 0.100 | 0.111 | 0.117 | 0.113 |

**Table S10. Correlations between maternal PFASs and fetal thyroid hormones adjusted by literature covariates.**  GW, gestation weeks. Spearman correlation tests were used among the concentrations of PFASs and thyroid hormones with adjustment influential covariates based on literature. Units in pg/mL for 6:2 FTS and NMeFOSAA, ng/mL for other PFASs, pmol/mL for FT3 and FT4, nmol/mL for T3 and T4, μIU/mL for TSH. Adjusted for maternal age, maternal prepregnancy BMI, neonatal sex, previous live births, neonatal type of delivery, with- or without gestation weeks. * *P* < 0.05, ** *P* < 0.01.

| **Fetal PFAS** | **Maternal FT3**  **No GW Adjusted** | | **Maternal FT4**  **No GW Adjusted** | | **Maternal T3**  **No GW Adjusted** | | **Maternal T4**  **No GW Adjusted** | | **Maternal TSH**  **No GW Adjusted** | |
| --- | --- | --- | --- | --- | --- | --- | --- | --- | --- | --- |
| PFOA | -0.161* | -0.160* | -0.087 | -0.090 | -0.047 | -0.043 | -0.057 | -0.059 | -0.070 | -0.106 |
| PFNA | -0.239** | -0.247** | -0.104 | -0.103 | -0.153 | -0.154 | -0.101 | -0.100 | -0.040 | -0.066 |
| PFDA | -0.247** | -0.252** | -0.119 | -0.119 | -0.210** | -0.205* | -0.120 | -0.115 | -0.133 | -0.145 |
| PFUnA | -0.286** | -0.290** | -0.073 | -0.062 | -0.200* | -0.198* | -0.043 | -0.036 | -0.143 | -0.144 |
| PFDoA | -0.247** | -0.239** | -0.088 | -0.089 | -0.210** | -0.209** | -0.123 | -0.125 | 0.040 | 0.047 |
| PFHxS | -0.163* | -0.163* | -0.015 | -0.011 | -0.154 | -0.165* | -0.037 | -0.035 | -0.086 | -0.111 |
| PFOS | -0.189* | -0.200* | -0.053 | -0.069 | -0.187* | -0.188* | -0.093 | -0.100 | -0.183* | -0.199* |
| 6:2 FTS | -0.129 | -0.117 | -0.021 | -0.019 | -0.078 | -0.073 | -0.072 | -0.068 | 0.129 | 0.132 |
| NMeFOSAA | 0.045 | 0.046 | -0.010 | -0.006 | -0.036 | -0.035 | -0.019 | -0.022 | 0.001 | 0.008 |

**Table S11. Correlations between fetal PFASs and maternal thyroid hormones adjusted by literature covariates.** GW, gestation weeks. Spearman correlation tests were used among the concentrations of PFASs and thyroid hormones with adjustment influential covariates based on literature. Four women with abnormal thyroid hormones levels were excluded. Units in pg/mL for 6:2 FTS and NMeFOSAA, ng/mL for other PFASs, pmol/mL for FT3 and FT4, nmol/mL for T3 and T4, μIU/mL for TSH. Adjusted for maternal age, maternal prepregnancy BMI, neonatal sex, previous live births, neonatal type of delivery, with- or without gestation weeks. * *P* < 0.05, ** *P* < 0.01.

| **Correlation test** | **Covariates selected from multivariate analysis** | **Covariates selected from multivariate literatures** |
| --- | --- | --- |
| Maternal PFASs & Maternal thyroid homones | maternal age, maternal prepregnancy BMI, maternal monthly income, and neonatal type of delivery (Table 4) | maternal age, gestation weeks, maternal prepregnancy BMI, previous live births, with or without maternal monthly income (Table S8) |
| Maternal PFASs & Fetal thyroid homones | maternal prepregnancy BMI, maternal monthly income, maternal previous live births and neonatal type of delivery (Table S2) | maternal age, maternal prepregnancy BMI, neonatal sex, previous live births, neonatal type of delivery, with or without gestation weeks (Table S9) |
| Fetal PFASs & Fetal thyroid homones | maternal prepregnancy BMI, maternal monthly income, neonatal birth length and neonatal type of delivery (Table S3) | maternal age, maternal prepregnancy BMI, neonatal sex, previous live births, neonatal type of delivery, with or without gestation weeks (Table S10) |
| Fetal PFASs & Maternal thyroid homones | maternal age, maternal prepregnancy BMI, maternal monthly income, maternal previous live births and neonatal type of delivery (Table 5) | maternal age, maternal prepregnancy BMI, neonatal sex, previous live births, neonatal type of delivery, with or without gestation weeks (Table S11) |

**Table S12.** **Comparation of covariates selected from multivariate analysis and literatures.**


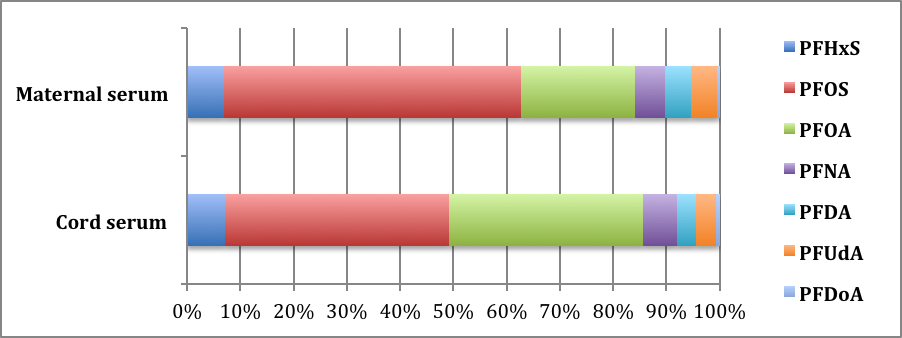


**Fig. S1. Composition profiles of major PFASs in maternal and cord serum.**
